# Towards patterned bioelectronics: facilitated immobilization of exoelectrogenic *Escherichia coli* with heterologous pili

**DOI:** 10.1111/1751-7915.13309

**Published:** 2018-09-17

**Authors:** Michael Lienemann, Michaela A. TerAvest, Juha‐Pekka Pitkänen, Ingmar Stuns, Merja Penttilä, Caroline M. Ajo‐Franklin, Jussi Jäntti

**Affiliations:** ^1^ VTT Technical Research Centre of Finland Ltd Espoo Finland; ^2^ Department of Biochemistry and Molecular Biology Michigan State University East Lansing MI USA; ^3^ The Molecular Foundry Lawrence Berkeley National Laboratory Molecular Biophysics and Integrated Bioimaging Division Synthetic Biology Institute Berkeley CA USA; ^4^ Current affiliation: Solar Foods Ltd Helsinki Finland

## Abstract

Biosensors detect signals using biological sensing components such as redox enzymes and biological cells. Although cellular versatility can be beneficial for different applications, limited stability and efficiency in signal transduction at electrode surfaces represent a challenge. Recent studies have shown that the Mtr electron conduit from *Shewanella oneidensis* MR‐1 can be produced in *Escherichia coli* to generate an exoelectrogenic model system with well‐characterized genetic tools. However, means to specifically immobilize this organism at solid substrates as electroactive biofilms have not been tested previously. Here, we show that mannose‐binding Fim pili can be produced in exoelectrogenic *E. coli* and can be used to selectively attach cells to a mannose‐coated material. Importantly, cells expressing *fim* genes retained current production by the heterologous Mtr electron conduit. Our results demonstrate the versatility of the exoelectrogenic *E. coli* system and motivate future work that aims to produce patterned biofilms for bioelectronic devices that can respond to various biochemical signals.

## Introduction

Extracellular electron transfer (EET) is a biochemical process during which electrons are transferred across the bacterial cell envelope (White *et al*., 2016). EET holds great potential for application in biosensing applications (Prévoteau and Rabaey, 2017) where EET‐performing cells represent an attractive alternative to less stable enzymes as sensing elements. Such biosensors contain the biological sensing element immobilized on a conductive surface where it performs the transduction of a specific chemical signal into an electric output. The capability of certain microbial species to reduce solid minerals at their surface allows their cultivation at the anode of a bioelectrochemical system (BES) and their genetic modification paved the way for their application in bioprocess and environmental monitoring (Webster *et al*., 2014; Prévoteau and Rabaey, 2017). In sensing applications, EET is utilized as a signal transducing process that, although slower than enzyme catalysts, is attractive when longevity, production costs, electric connection of the biological component, sensitivity and simultaneous processing of several signals are important.

**Figure 1:**
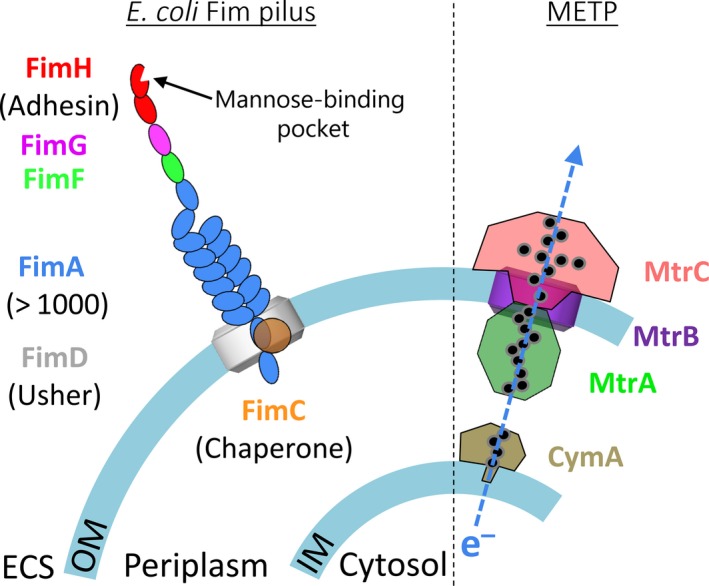
Schematic of cell envelope of the constructed *E. coli* system. Fim pili are attached to the outer membrane (OM) and *S. oneidensis* MR‐1 Mtr electron transfer pathway (METP) components are associated with outer and inner membrane (IM). Electron‐conducting haem cofactors are displayed as filled black circles. The mechanism through which electrons are exchanged between IM‐bound CymA and OM‐bound MtrA is currently not known, but it is speculated that MtrA may participate in periplasmic electron transfer based on the observation of MtrA in the periplasmic compartment (Jensen *et al*., 2016).

Three decades ago, EET was discovered as manganese and iron respiration in species of *Geobacter* and *Shewanella* (Lovley and Phillips, 1988; Lovley, 1991; Marsili *et al*., 2008a). More than 90 species have been reported to perform EET (Koch and Harnisch, 2017) including the EET model organisms *Geobacter sulfurreducens* and *Shewanella oneidensis*. Both species gain chemical energy in the form of ATP by coupling carbon oxidation to anaerobic reduction of metal oxide minerals such as Mn(III), Mn(IV), Fe(III), Cr(VI) and U(IV) in the extracellular space (Richter *et al*., 2012). Despite the considerable number of reported electroactive microbes, the composition of their extracellular electron transfer pathway has remained largely unknown and is still limited to a small number of anode‐respiring bacteria (Kracke *et al*., 2015). In *S. oneidensis*, EET is initiated at the inner cell membrane where electrons, originating from the oxidation of primary fermentation products (e.g. lactate, pyruvate, formate and hydrogen), are transferred to membrane‐embedded menaquinols by formate and NADH dehydrogenases (Meshulam‐Simon *et al*., 2007; Pinchuk *et al*., 2009; Cordova *et al*., 2011; Brutinel and Gralnick, 2012a; Kane *et al*., 2016). Menaquinol is in turn oxidized by the inner membrane‐bound quinol dehydrogenase CymA (Marritt *et al*., 2012), or in its absence, the enzyme complex SirCD (Cordova *et al*., 2011). Next, electrons are transferred across the periplasmic space by diffusible *c*‐cytochromes STC and FccA (Schuetz *et al*., 2009; Fonseca *et al*., 2013) and transferred to terminal reductases at the outer membrane such as MtrA and MtrC which are associated with the MtrCAB complex (Fig. 1) (Coursolle and Gralnick, 2010; Breuer *et al*., 2015). On the extracellular face of the outer membrane, MtrC accepts electrons from MtrA and transfers these charges either directly onto an insoluble electron acceptor or onto secreted flavin molecules (Brutinel and Gralnick, 2012b; Xu *et al*., 2016).

Several research groups have used current knowledge on extracellular respiration to engineer non‐natural exoelectrogens by synthetic biology methods (TerAvest and Ajo‐Franklin, 2016). Among these, the transfer of the EET capacity of *S. oneidensis* into the well‐studied gene expression host *E. coli* has been explored in several studies (Pitts *et al*., 2003; Gescher *et al*., 2008; Jensen *et al*., 2010; Goldbeck *et al*., 2013; TerAvest *et al*., 2014; Sturm‐Richter *et al*., 2015; Förster *et al*., 2017). One such EET system was developed recently by Jensen *et al*., who have incorporated the *S. oneidensis* inner membrane *c*‐cytochrome CymA and the outer membrane complex MtrCAB into *E. coli* yielding the strain *cymAmtrCAB* (Jensen *et al*., 2016). Remarkably, this strain was able to transfer electrons across the cell envelope in the absence of the periplasmic *S. oneidensis* cytochromes STC and FccA. Since CymA can reduce MtrA *in vitro* (Firer‐Sherwood *et al*., 2011), Jensen *et al*. proposed that the electrons transit the ~15 nm wide *E. coli* periplasm (Matias *et al*., 2003) by diffusion of periplasmic MtrA. Hence, the four *S. oneidensis* MR‐1 proteins CymA and MtrCAB form a functional Mtr electron transfer pathway (METP) in *E. coli* and were employed in the present study as a membrane‐bound electron conduit. Electroactive *E. coli* strains such as *cymA‐mtrCAB* differ from naturally occurring electroactive species in that the currently available exoelectrogenic *E. coli* strains neither form cohesive biofilms on electrodes nor utilize efficient extracellular electron transport mediators such as conductive pili or flavins. When considering strict anaerobic exoelectrogenic species such as *G. sulfurreducens*, the oxygen tolerance of *E. coli* can be regarded as a desirable feature as it may facilitate its incorporation in novel type of bioelectronics that are usually handled under ambient conditions. In addition, the use of non‐biofilm‐forming cells in electrochemical biosensors is complicated by complex chemical or physical immobilization and, in the case of biofilm‐forming species, low reproducibility, required initial growth time and limited strain availability have to be considered during sensor design (Prévoteau and Rabaey, 2017).

In order to further increase the relevance of the electroactive biological cell to bioelectronic applications, it is necessary to develop strains that can be stably and selectively immobilized at conductive substrates *in situ*. This may be done by, for instance, patterning of a biosensor surface with a synthetic biofilm consortium that can detect and process multiple signals in parallel. The attachment of current‐producing bacteria on conductive surfaces was attempted previously using gold‐binding peptides that were integrated with an *E. coli* outer membrane protein and displayed on the outer surface of *S. oneidensis* (Kane *et al*., 2013). This system failed to maintain current production while introducing binding and, thereby, no strategy is currently available for achieving patterning with exoelectrogenic bacteria. The specific and strong attractive interactions that are required for such an attachment are naturally provided by rod‐like carbohydrate‐binding cell appendages called pili (Fig. 1). These protein structures are naturally produced by, for example, uropathogenic *E. coli* strains (Thomas *et al*., 2004; Korea *et al*., 2011) and are potentially useful additions to the exoelectrogenic *E. coli* strain because these appendices have naturally evolved to mediate attachment with low impact on other outer membrane functions. Here, we explore the possibility of obtaining an exoelectrogenic biofilm‐forming *E. coli* variant by production of mannose specific Fim pili and report the effect of pili production on the cytochrome production levels, the exoelectrogenic performance and its adhesive properties with respect to Fim ligands and an electrode surface.

## Results and discussion

### Design of *E. coli* coproducing CymAMtrCAB and FimAICDFGH for the biochemical characterization of Fim pili and the METP

In order to test whether functional Fim pili and the METP comprising the *S. oneidensis* electron transfer proteins CymA and MtrCAB can be coproduced in *E. coli*, a set of four *E. coli* strains was constructed (Table 1). This included a reference strain, called *cymA*‐*mtr*‐Sm^R^, which was prepared from the existing exoelectrogenic *E. coli* strain *cymA‐mtr* (TerAvest *et al*., 2014) by transformation with the insert‐free expression vector pCDFDuet‐1. In addition to pCDFDuet‐1, this *E. coli* strain contained the vectors pEC86 and I5049, which are required for the efficient production of the *S. oneidensis* electron conduit in *E. coli*. The recombinantly expressed genes comprised the *E. coli* cytochrome *c* maturation genes *ccmA‐H*, which were incorporated in the pEC86 construct under the control of the constitutive *tet* promoter. The *cymAmtrCAB* gene cluster was placed downstream of the IPTG‐inducible T7/*lac* promoter in plasmid I5049. Three additional *E. coli* strains were prepared in order to investigate whether functional Fim pili can be produced in the studied *E. coli* system and distinguished from the non‐piliated phenotype. These strains were designed to either coproduce Fim pili and CymAMtrCAB (strain *cymA*‐*mtr*‐*fim*), to exclusively produce Fim pili as heterologous membrane protein (strain *fim*‐Kan^R^) or not to produce any heterologous cell envelope proteins at all (strain Kan^R^‐Sm^R^). All these strains carried three different plasmids of which the *ccmA‐H* expression construct pEC86 was included in every strain. In addition, Kan^R^‐control strains Kan^R^‐Sm^R^ and *fim*‐Kan^R^ contained the empty pET30a+ derivative pSB1ET2 while the strains *cymA‐mtr*‐Sm^*R*^ and *cymA‐mtr*‐*fim* carried the pSB1ET2 derivative I5049 with added *cymAmtrCAB* gene insert under the control of the T7/*lac* promoter. Non‐piliated control strains (Sm^R^) harboured the insert‐free expression construct pCDFDuet‐1 and piliated *fim* strains contained the pCDFDuet‐1 derivative pMil20. This plasmid included the Fim pili gene cluster *fimAICDFGH* inserted downstream of the IPTG‐inducible T7/*lac* promoter.

**Table 1:** Bacterial strains produced in the present study. All strains are derivatives of the *E. coli* cell line C43(DE3)

*E. coli* strain	Plasmids^a^	VTT Culture collection ID
Kan^R^‐Sm^R^	pEC86 (*ccmA‐H*), pSB1ET2 (‐), pCDFDuet‐1 (‐)	E‐183553
*cymA*‐*mtr*‐Sm^R^	pEC86 (*ccmA‐H*), I5049 (*cymAmtrCAB*), pCDFDuet‐1 (‐)	E‐183554
*fim*‐Kan^R^	pEC86 (*ccmA‐H*), pSB1ET2 (‐), pMil20 (*fimAICDFGH*)	E‐183555
*cymA*‐*mtr*‐*fim*	pEC86 (*ccmA‐H*), I5049 (*cymAmtrCAB*), pMil20 (*fimAICDFGH*)	E‐183556

**a**. Inducible genes stated in brackets with ‘‐’ denoting an insert‐free vector.

### Recombinant Fim pili production attenuates CymA concentration but imposes little stress on exoelectrogenic *E. coli*

**Figure 2:**
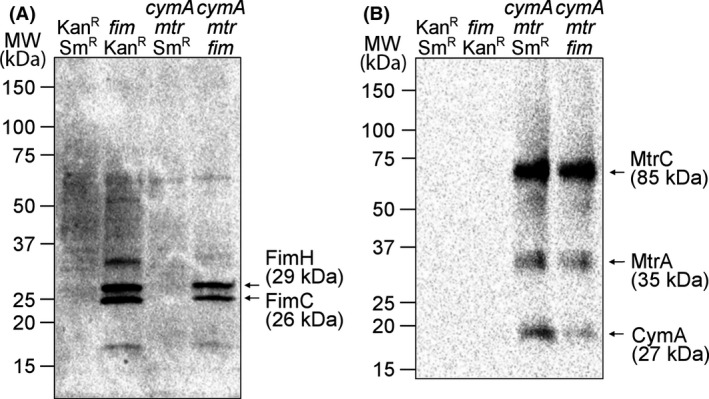
Analysis of recombinant protein levels in *E. coli* strains using SDS‐PAGE (4–20% polyacrylamide gradient) and Western blot analysis. The analysed cell lysates were prepared from strains that either did not contain *S. oneidensis* EET components or pili genes (Kan^R^‐Sm^R^), the Fim pili gene cluster *fimAICDFGH* but no encoded *S. oneidensis* EET components (*fim*‐Kan^R^), no Fim pili genes but the *S. oneidensis* EET component gene cluster *cymAmtrCAB* (*cymA*‐*mtr*‐Sm^R^) or both, pili and *S. oneidensis* EET component gene clusters (*cymA*‐*mtr*‐*fim*). The cells were collected following an overnight incubation in a BES at a *E*_WE_ of +200 mV versus Ag/AgCl. The separated cell lysates were labelled with rabbit antibodies specific for Fim pili components FimC and FimH (A). Bound antibodies were visualized by enhanced chemiluminescence following addition of horseradish peroxidase‐conjugated secondary antibodies (A). In subfigure B, redox‐active cytochromes CymA, MtrA and MtrC were detected directly by enhanced chemiluminescence. The positions of molecular weight standard proteins, the predicted positions of the antibody labelled proteins (A) and haem‐containing proteins (B) along with their molecular weights are indicated.

The heterologous production of recombinant outer membrane proteins in the host *S. oneidensis* has been reported to adversely affect the production of the outer membrane protein complex MtrCAB (Kane *et al*., 2013). To test whether this is also true in the case of added Fim pili in the exoelectrogenic *E. coli* strains, we cultivated the four *E. coli* strains Kan^R^‐Sm^R^, *fim*‐Kan^R^, *cymA*‐*mtr*‐Sm^R^ and *cymA*‐*mtr*‐*fim* in BES's under anaerobic conditions overnight at a working electrode potential of +200 mV versus Ag/AgCl. Samples were taken from the BES and analysed with regard to the cell content of type I pili components using immunoblotting (FimCH) and electron transfer proteins using enhanced chemiluminescence (CymA, MtrA and MtrC). A comparison of the FimC and FimH production levels in the whole cell lysates of two *fim* strains revealed that intensity of both proteins was lower when the METP genes were coexpressed (Fig. 2A). Similarly, analysis of the *c*‐type cytochromes revealed that CymA levels were clearly decreased while MtrC and MtrA levels were largely unchanged when compared to the strain lacking the *fim* gene cluster (Fig. 2B). We speculate that the decrease in relative expression levels is due to a limited overall production capacity for membrane proteins. The stress imposed on the exoelectrogenic *E. coli* cell by recombinantly produced pili and electron transfer proteins was monitored through the rate of growth at 37°C. These experiments showed that there was no measurable difference in growth rate between the control strain Kan^R^‐Sm^R^ and the Fim pili gene expressing strain *fim*‐Kan^R^ (Appendix S1). In the presence of cytochrome genes, a 20% decreased growth rate was measured when compared to the *E. coli* strains lacking the METP genes. These data indicate that the production of the *S. oneidensis* electron transfer components is a larger energetic burden to the *E. coli* cell than the production of *E. coli* Fim pili.

### Fim gene expression increases binding to mannose‐coated surfaces

**Figure 3:**
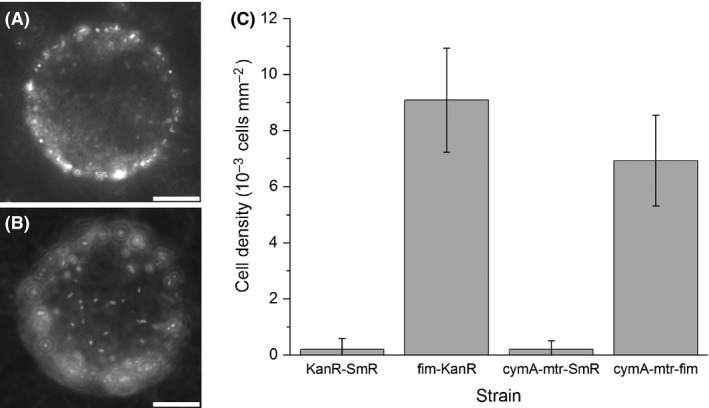
Mannose‐binding assay investigating binding of the *E. coli* strains Km^R^‐Sm^R^, *fim*‐Kan^R^, *cymA*‐*mtr*‐Sm^R^ and *cymA*‐*mtr*‐*fim* to mannose‐coated agarose beads. A dense corona of propidium iodide‐stained *E. coli fim*‐Kan^R^ cells is apparent at the bead centre focus and at the top pole focus (A and B, respectively; 20 μm scale bar). A comparison of cell binding as determined from top bead poles (C) reveals that both pili‐producing strains *fim*‐Kan^R^ and *cymA*‐*mtr*‐*fim* bound to the mannose beads at high densities of > 5 × 10^3^ cells mm^−2^ while no significant attachment was measured with strains Km^R^‐Sm^R^ and *cymA*‐*mtr*‐Sm^R^. For each strain, the density of bound cells was determined from five beads with similar diameter of around 100 μm. The imaged spherical cap area was determined from the bead diameter and the assayed projected cap area.

The mannose specific binding of the *E. coli* strains was studied as adsorption to mannose‐functionalized agarose beads. Microscopy images reveal attached cells in the case of *fim* strains as apparent for strain *fim*‐Kan^R^ (Fig. 3A and B). The variant *cymA*‐*mtr*‐*fim* formed slightly less dense cell layers, while almost no attachment of cells was observed in the case of the non‐piliated control strains Kan^R^‐Sm^R^ and *cymA*‐*mtr*‐Sm^R^ (Fig. 3C). These experiments showed that mannose‐binding Fim pili can be produced in the exoelectrogenic *E. coli* system and that the two *E. coli fim* strains form biofilms on mannose with no significant background binding.

### The capacity of the METP to reduce soluble substrates is preserved during pili coproduction

**Figure 4:**
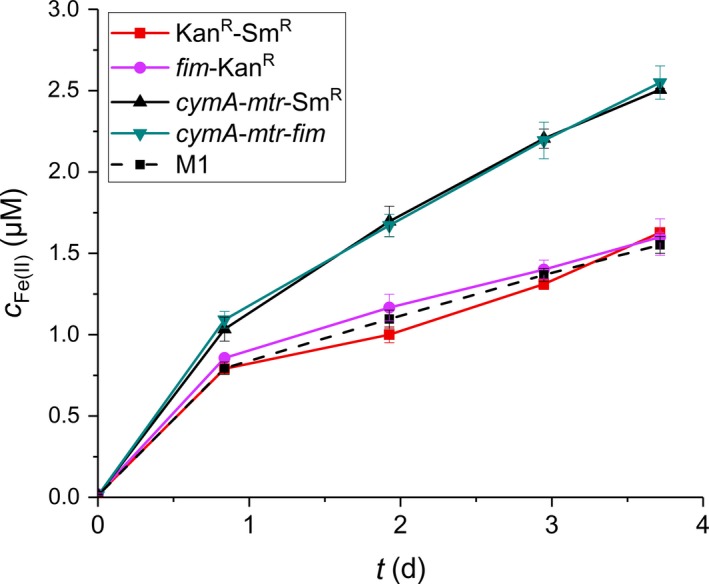
Ferric iron reduction measured by the ferrozine method in M1 medium without cells (M1) and in the presence of cells of *E. coli* strains Kan^R^‐Sm^R^, *fim*‐Kan^R^, *cymA*‐*mtr*‐Sm^R^ and *cymA*‐*mtr*‐*fim*. The M1 medium was supplemented with 10 mM Fe(III) citrate and 40 mM lactate. During overnight incubation prior to supplement addition, the bacterial cells were exposed to an *E*_WE_ of +200 mV versus Ag/AgCl. Displayed are averages of triplicate measurements with the corresponding standard deviation as error bars.

The effect of pili coproduction on the Mtr electron transfer functionality was measured using a soluble Fe(III) reduction assay. This was done using cells of all four *E. coli* strains that were, prior to this reduction assay, incubated overnight in an anaerobic BES at a working electrode potential of +200 mV versus Ag/AgCl. The observed Fe(II) formation indicated that the two *cymA*‐*mtr* strains were performing the Fe(III) reduction at identical rates that were significantly above the apparent background reduction activity as measured in M1 medium (Fig. 4). The Fe(III)‐reductive activity of both Kan^R^‐control strains was identical to the background activity, which confirmed that this assay specifically measures the activity of the METP. Based on the differential CymA production (Fig. 2B), the unchanged ferric citrate reduction rate of *cymA*‐*mtr*‐*fim* when compared to the non‐piliated strain *cymA*‐*mtr*‐Sm^R^ is an unexpected result. The apparent tolerance of the exoelectrogenic *E. coli* to decreased CymA levels may be due to the presence of the native inner membrane cytochrome NapC which has been proposed to fulfil the same EET function as CymA (Jensen *et al*., 2016). These data suggest that Fim pili and the METP can be coproduced without affecting the capability of the cell to reduce soluble electron acceptors.

### *E. coli* producing type 1 pili and the METP produce current and show increased persistence in BESs

**Figure 5:**
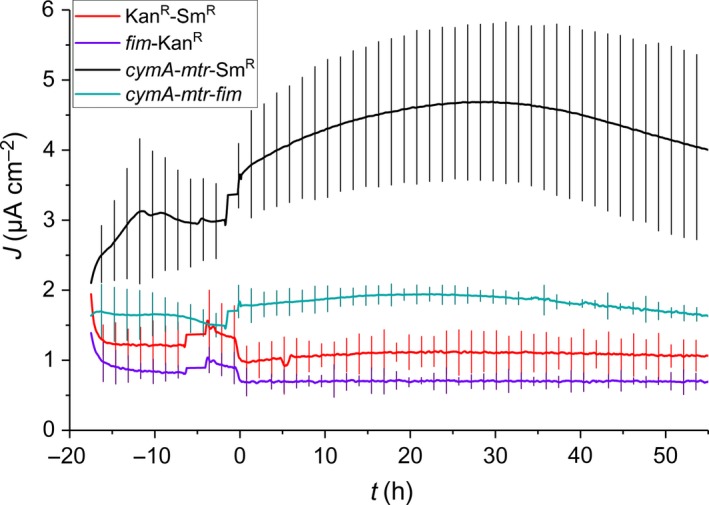
Chronoamperometry analysis of *E. coli* strains Kan^R^‐Sm^R^, *fim*‐Kan^R^, *cymA*‐*mtr*‐Sm^R^ and *cymA*‐*mtr*‐*fim* interacting with a carbon cloth working electrode poised at *E*_WE_ = +200 mV versus Ag/AgCl. Lactate was added at *t* = 0 h to a final concentration of 40 mM following an 18‐h precultivation without carbon source. Error bars indicate standard error of triplicate measurements.

Lactate cannot be fermented by *E. coli* and was therefore chosen as a substrate to test the ability of the cell to transfer electrons released from substrate oxidation to an extracellular electrode. The current response was monitored by chronoamperometry for at least 50 h following addition of lactate [40 mM]. The initial lactate concentration was chosen based on previous studies in which the exoelectrogenic *E. coli* system produced a clear electric signal under similar conditions (TerAvest *et al*., 2014). The cytochrome‐free strains *fim*‐Kan^R^ and Kan^R^‐Sm^R^ produced constant currents throughout the measured interval with current densities of 0.70 ± 0.08 and 1.09 ± 0.07 μA cm^−2^ respectively (Fig. 5). Those background currents may be related to hydroquinone derivatives that have previously been proposed to be produced by *E. coli* cells and which may act as mediators of extracellular electron transport (Qiao *et al*., 2011). The highest electroactivity was measured with the cytochrome producing strain *cymA*‐*mtr*‐Sm^R^ that started with a current density of 3.6 μA cm^−2^ followed by a steady increase to a maximum current density of 4.5 μA cm^−2^ over a 28 h period after which the current decreased to 4.0 μA cm^−2^ by the end of the experiment. The current density produced by the strain which coproduced pili and cytochromes increased from 1.8 μA cm^−2^ to a maximum of 1.9 μA cm^−2^ during 20 h and decreased to a level of 1.7 μA cm^−2^ at 55 h past lactate addition. Monitoring of the lactate concentration by HPLC analysis showed that all tested strains consumed only a small fraction of the added lactate substrate during the electrocultivation, which is consistent with predictions based on the observed electric currents and showed that the availability of this substrate was not limiting metabolic activity. The current produced by strain *cymA*‐*mtr*‐*fim* was significantly higher than the current of 1.07 μA cm^−2^ that was measured with the most electroactive non‐Mtr control strain Kan^R^‐Sm^R^ (Fig. 5, Appendix S2; *P *=* *0.018; two‐tailed Student's *t*‐test).

Interestingly, the current produced by *E. coli cymA*‐*mtr*‐*fim* was 58% lower than the electric current measured with the non‐piliated form (i.e. *cymA*‐*mtr*‐Sm^R^: 4.0 μA cm^−2^). The ferric citrate reduction assay has shown that this current decrease is not due to a METP disruption. We speculate that this effect is due to steric restrictions of attractive electrode–cell interactions that are imposed by Fim pili and lead to a reduction in outer membrane cytochromes (such as MtrC) that are close enough to the electrode surface to participate in direct electron transfer. This hypothesis is supported by the observation that cell suspensions of both *fim* strains do not sediment upon static incubation at room temperature whereas both Sm^R^ strains settle out of the fluid under these conditions (data not shown). A similar phenomenon has been reported by Meuskens *et al*. (2017) who have observed increased flocculation of *E. coli* strains which lacked several outer membrane proteins. As an explanation, the authors proposed a shielding function of outer membrane proteins that controls aggregation by limiting electrostatic attraction between cells. Such charge shielding may also limit electron transfer to the carbon electrode in the exoelectrogenic *E. coli* system but does, as is apparent from the unchanged ferric iron reduction, not affect the electron transfer to soluble electron acceptors. The small size of the ferric iron anion may allow it to penetrate the pili layer and support EET in pili‐producing cells at the same rate as in non‐piliated cells. The shielding effects of pili may be mitigated by usage of a more tunable induction system for pili gene expression.

Exoelectrogenic *E. coli* cells spontaneously adsorb to carbon electrodes as thin layers and since type I pili promote the aggregation of *E. coli* cells (Ogden and Taylor, 1991), we hypothesized that the thickness of a spontaneously adsorbed exoelectrogenic biofilm could be increased by expression of *fim* genes. To test this, we measured the biomass present in solution and on the anode surface after 5 days in the BESs. Examination of anode‐bound biomass and biomass in solution shows that, while there are no significant differences in the solution biomass among the different strains, there is a significant ~2.5‐fold increase in anode‐bound biomass in the *cymA*‐*mtr*‐*fim* strain relative to the strains Kan^R^‐Sm^R^ and *fim*‐Kan^R^ (10.28 ± 1.66 mg versus 3.69 ± 0.50 mg and 3.31 ± 0.21 mg, *P *=* *0.003 and 0.002, respectively; two‐tailed Student's *t*‐test; comparison presented in Appendix S2). This observation indicates that, unlike adhesion to mannose‐coated surfaces, *fim* expression alone was insufficient to significantly increase cell biomass on the anode. Rather, both Fim pili and the METP were required to enhance cell binding to the anode.

The exact mechanism through which the METP promotes cell adhesion is not evident from the presented data but may be linked to the cellular anodic respiration, which can only occur while the bacterial cell is in contact with the electrode. The Kan^R^ strains exhibited 40% background adsorption to carbon cloth, whereas no significant background adhesion was measured with the strains *cymA*‐*mtr*‐Sm^R^ and Kan^R^‐Sm^R^ on mannose. This indicates that utilization of Fim pili based carbohydrate binding for immobilization of exoelectrogenic cells is preferable over cytochrome mediated adsorption to carbon and a promising approach to achieve specific binding at solid surfaces. For this approach, the carbon electrode has to be modified with mannose groups which has been achieved previously by carbodiimide coupling of amino‐functionalized mannose to a carboxylated carbon electrode substrate (Gu *et al*., 2008; Ehlert *et al*., 2011). Carbohydrates may also be immobilized on gold (He *et al*., 2011), which, based on its low resistance and inertness, appears as an interesting alternative to carbon in biosensing applications. However, gold is prone to spontaneous protein adsorption (De Paoli Lacerda *et al*., 2010), which may prevent specific cell adhesion and external electron transfer.

### Coproduction of the METP and cell adhesion proteins in *E. coli* offers new opportunities for bioelectronic applications

The presented exoelectrogenic *E. coli* strain may be developed further for bioelectronic applications that demand both, specific surface binding and higher EET rate by introduction of genetic modifications that result in a lower pili density and thereby improve the electron transfer through the extracellular space. Furthermore, the EET efficiency of this strain could be improved by utilization of the currently known conductive pili (Holmes *et al*., 2016) by, for instance, coproduction of conductive and adhesive pili in the same cell or by replacing the Fim pili with novel rationally designed hybrid pili that consist of a long conductive fibre and a terminal carbohydrate‐binding adhesin. Alternatively, the EET of *E. coli cymA*‐*mtr*‐*fim* could be promoted through penetration of the pili layer with electrode‐tethered conductive polymers such as linear naphtoquinone‐modified polyethylenimine hydrogels through which electrons can be transferred by shuttling between the covalently bound redox centres (Milton *et al*., 2015).

Among the selective cell adhesion systems found in nature, the adhesin/lectin–carbohydrate system is most common and includes a wide range of ligand–receptor pairs. In addition to this approach, other strategies have been employed, like, for example, biofilm patterning with a genetically engineered *E. coli* strain that adsorbed to various substrates with Ag43 adhesin upon illumination (Jin and Riedel‐Kruse, 2018). The Ag43 adhesin system is, however, considered disadvantageous for the proposed signal multiplexing because it is non‐specific and therefore incompatible with selective binding of different types of cells from a mixture. Other cell adhesion systems with technical relevance include, the cell binding by cell‐surface bound antibody targets (Suo *et al*., 2008) and recombination of protein dimer components exposed at the outer cell membrane and the adhesion substrate (Chen and Wegner, 2017).

Existing electroactive biosensing applications, relevant for the exoelectrogenic *E. coli* system, include predominantly multispecies systems that were designed for monitoring of process parameters such as the oxygen demand, the production of volatile fatty acids and presence of toxins (Modin and Aulenta, 2007). In addition, several single‐species sensing system have been developed for, for example, monitoring of acetate by *G. sulfurreducens* (Tront *et al*., 2008; Nevin *et al*., 2011), sensing of lactate, fumarate and arabinose by *S. oneidensis* (Kouzuma *et al*., 2012; Golitsch *et al*., 2013; Si *et al*., 2015) and the detection of arsenic by *Enterobacter cloacae* and *S. oneidensis* (Webster *et al*., 2014; Rasmussen and Minteer, 2015). More advanced applications include the cell‐internal processing of quorum sensing signals which has been achieved by the creation of AND logic gates in *S. oneidensis* and *P. aeruginosa* (Li *et al*., 2011; Hu *et al*., 2015). The performance of biosensors will benefit from miniaturization, for example, sensing in microfluidic systems. The coproduction of METP and adhesive pili is a novel strategy with which electroactive cells can be targeted to inorganic surfaces and is therefore a promising approach for the realization of such patterned bioelectronics.

## Experimental procedures

### Strains and plasmids

Information on the plasmids, bacterial strains and primers used in this study is given in Table 1 and Appendix S3–S5. The plasmid pMil020 for recombinant Fim pili production was constructed by subcloning the *fim* operon as two PCR fragments from pSH2 (courtesy of Prof. Paul Orndorff) (Orndorff and Falkow, 1984). The *fimAICD* gene fragment was amplified and extended with a 5′‐terminal restriction site using the PCR primers NdeI_fim_fw1 and SH2_27_rv. The *fimDFGH* gene fragment was amplified with a 3′‐terminal *Avr*II restriction site using the primers SH2_24_fw and AvrII_fimH_rv1. Both fragments were cloned into pCR2.1‐TOPO vectors (*fimAICD*: pCR2.1‐TOPO+PCR4‐c6; *fimDFGH*: pCR2.1‐TOPO+PCR5‐c6) using the TOPO TA cloning kit (Thermo Fisher Scientific, Waltham, MA, USA) and confirmed by sequencing. The vectors pCR2.1‐TOPO+PCR4‐c6 and pCR2.1‐TOPO+PCR5‐c6 were subjected to *Nde*I–*Bam*HI and *Bam*HI–*Avr*II digest to yield *fimAICD* and *fimDFGH* fragments respectively. The vector pCDFDuet‐1 was double‐digested using restriction enzymes *Nde*I and *Avr*II, and the resulting large vector fragment was ligated with the purified *fimAICD*‐ and *fimDFGH* fragments yielding the expression construct pMil020 (Appendix S3 and S4). The correct assembly was confirmed by sequencing. An *E. coli* XL‐1 Blue strain harbouring the plasmid pMil20 was deposited with the VTT Culture Collection (strain ID E‐183557) as well as the presented *E. coli* C43(DE3) strain variants Kan^R^‐Sm^R^ (strain ID E‐183553), *fim*‐Kan^R^ (strain ID E‐183555), *cymA*‐*mtr*‐Sm^R^ (strain ID E‐183554) and *cymA*‐*mtr*‐*fim* (strain ID E‐183556) (see Table 1).

### Culture conditions

Prior to BES cultivation, bacterial cells were grown overnight at 37°C and 220 rpm in 5 mL 2×YT broth (16 g l^−1^ tryptone, 10 g l^−1^ yeast extract, 5 g l^−1^ NaCl, pH 7.0, supplemented with 50 μg ml^−1^ kanamycin, 100 μg ml^−1^ chloramphenicol and 50 μg ml^−1^ streptomycin). Then, 500 μl preculture was added to 50 ml 2×YT broth and incubated at 30°C and 220 rpm until an OD_595 nm_ of 0.5–0.9 was reached. The inducer isopropyl *β*‐d‐1‐thiogalactopyranoside (IPTG) was then added to a final concentration of 50 μM. The production of recombinant proteins was allowed for about 20 h at a reduced agitation of 200 rpm. Cell cultures harbouring the I5049 vector were supplemented with the haem precursor *δ*‐aminolevulinic acid at a final concentration of 1 mM in order to enhance haem biosynthesis and optimal cytochrome production (Fernandes *et al*., 2008).

### Detection of Fim pili component and cytochrome production by Western blot analysis

Frozen *E. coli* cell samples were thawed and resuspended in B‐PER bacterial protein extraction reagent (Thermo Fisher Scientific) at an OD_595 nm_ of 3.5. The following components were added at the given final concentrations: 1.0 mM EDTA, 4.2 mM MgCl_2_, 12 μg ml^−1^ chicken egg lysozyme, 10 μg ml^−1^ DNase I and 1.0 mM PMSF. The lysis reaction was performed at room temperature for 1 h followed by separation of the protein components in the lysed sample by reducing (analysis of Fim pili components) or non‐reducing (analysis of cytochromes) SDS‐PAGE (4–20% polyacrylamide gradient). The separated proteins were blotted onto nitrocellulose. Pili components FimC and FimH were labelled with a FimCH specific rabbit primary antibody (courtesy of David G. Thanassi) at a working dilution of 1:50 000. Primary antibody binding was detected by enhanced chemiluminescence using a rabbit‐IgG‐(H+L) specific antibody that was conjugated to horseradish peroxidase (Rockland Immunochemicals, Limerick, PA, USA) and reacted with SuperSignal West Pico chemiluminescent substrate (Thermo Fisher Scientific). The cytochrome detection was carried out by exposing the nitrocellulose membrane directly to the chemiluminescent substrate as previously described by Vargas *et al*. (1993).

### Mannose‐binding assay

Functional Fim pili production was monitored using a mannose‐binding assay. *E. coli* cultures in which recombinant gene transcription was induced with IPTG (see above) were collected by centrifugation (15 min at 4°C and 3200 *g*) and resuspended in M1 medium (see Appendix S6). d‐mannose‐coated agarose beads (Ø = 52–165 μm; bioWORLD, Dublin, OH, USA) were washed twice with ddH_2_O and thrice with M1 medium (seven bead volumes, each). Washed beads (V = 5.5 μl) were combined with the cell suspension in microtitre plate wells and diluted with M1 medium to OD_595 nm_ = 0.2 (V_Total_ = 200 μl per well). The plate was sealed and incubated for 1 h at 37°C followed by sedimentation of the beads for 5 min at 22°C and 400 *g*. The supernatant was replaced with sterile M1 medium and the plate was incubated overnight at 22°C. Bacterial cells were fluorescently stained by addition of 0.3 μl propidium iodide solution (20 mM in DMSO) to each microtitre plate well and a 15‐min incubation at 22°C in the dark. The reaction liquid was replaced with M1 medium and cell binding examined at λ_Ex_ = 520–550 nm using an IX81 inverted microscope (Olympus, Tokyo, Japan).

### Soluble Fe(III) reduction assay

All preparative steps of this method were performed in an anaerobic cabinet (80% N_2_, 10% CO_2_, 10% H_2_). Cells suspended in M1 medium containing lactate [40 mM] were diluted to OD_595 nm_ = 0.5 using the same medium. Fe(III) citrate was added to the cells at a final concentration of 10 mM and the Fe(II) concentration was monitored by daily sampling. Fe(II) in 50 μl aliquots was acid extracted for 1 h in 0.5 mL 0.5 M HCl. The Fe(II) concentration was determined using a modified method from L. Stookey (Stookey, 1970) according to which 50 μl of the acid extract was added to 1.0 mL ferrozine solution (c = 1.0 M; dissolved in 0.1 M HEPES, pH 8.0) and left to react for 10 min. The concentration of the Fe(II)–(ferrozine)_3_^4−^ complex was determined from A_563 nm_ (ε_563 nm_ = 27.9 mM^−1^ cm^−1^) of 200‐μl reaction volumes using a Varioskan microtitre plate reader (Thermo Fisher Scientific).

### BES setup

The voltammetry cells were assembled according to the setup described earlier by Marsili *et al*. (2008b) with slight modifications. In detail, the setup comprised a 6.4‐cm^2^‐sized rectangular carbon cloth working electrode (Panex 30–SW08; Zoltek, Nyergesújfalu, Hungary) which was cleaned by incubation in 1 M NaOH, 1 M HCl, acetone (2×) and ddH_2_O (2×) and attached to a custom‐made polyether ether ketone barrel (Ø = 9 mm) using Pt wire (Ø = 0.05 mm). The cell lid was manufactured in‐house from Teflon, and the remaining components were purchased from Bioanalytical systems (West Lafayette, IN, USA) including a MR‐1194 voltammetry cell, a MW‐4130 Pt wire counter electrode (Ø = 0.05 mm) and an MF‐2079 Ag/AgCl reference electrode that was contained in a MF‐2031 glass chamber, capped with a MF‐2064 Vycor frit and filled with 0.1 M NaCl. The assembled BES was sterilized by autoclaving without the Ag/AgCl reference electrode. After growth, the IPTG‐induced cells were collected by centrifugation (2450 *g*, 15 min, 4°C) and washed three times in 20 mL ice‐cold M1 medium. The cells were resuspended in 10 mL and diluted to OD_595 nm_ = 1.0 in a final volume of 60 mL using M1 medium as diluent. The remaining cells were collected by centrifugation (2450 *g*, 15 min, 4°C) and stored as a frozen pellet for Western blot analysis. For electrochemical measurements, 60 mL cell suspension was transferred into each BES, stirred at 200 rpm, maintained at 30°C and sparged with a humidified anoxic atmosphere (80% N_2_, 20% CO_2_) at a flow rate of 5 ml min^−1^.

### Electrochemical measurements

Chronoamperometry experiments were executed using a WaveNow potentiostat (Pine Research Instrumentation, Durham, NC, USA).

## Conflicts of interests

We declare that there are no conflicts of interests regarding the publication of this manuscript.

## Supporting information


**Appendix S1**. Effect of the introduced recombinant genes on the growth of the tested *E. coli* strains.
**Appendix S2**. Biomass distribution and current density determined from BES cultivations of *E. coli* strain set.
**Appendix S3**. Plasmids used in this study.
**Appendix S4**. Map of vector pMil020.
**Appendix S5.** Primers used in this study.
**Appendix S6.** Composition of M1 medium.
