# Supplementary material for: Towards patterned bioelectronics: facilitated immobilization of exoelectrogenic Escherichia coli with heterologous pili
Source: Microb Biotechnol. 2018 Sep 17;11(6):1184–94. doi: 10.1111/1751-7915.13309 (PMC6196383; doi:10.1111/1751-7915.13309)
Supplement: Supplementary file 1 — Appendix S1. Effect of the introduced recombinant genes on the growth of the tested E. coli strains. Appendix S2. Biomass distribution and current density determined from BES cultivations of E. coli strain set. Appendix S3. Plasmids used in this study. Appendix S4. Map of vector pMil020. Appendix S5. Primers used in this study. Appendix S6. Composition of M1 medium. [file MBT2-11-1184-s001.docx]

**Supporting information**

(Associated with manuscript “Towards patterned bioelectronics: Facilitated immobilization of exoelectrogenic *Escherichia coli* with heterologous pili” by Michael Lienemann, Michaela A. TerAvest, Juha-Pekka Pitkänen, Ingmar Stuns, Merja Penttilä, Caroline M. Ajo-Franklin and Jussi Jäntti)

**Appendix S1: Effect of the introduced recombinant genes on the growth of the tested *E. coli* strains**

The displayed data are averages of triplicate experiments. Slopes of the fitted regression curves are 0.352 1/h (*fim*-Kan^R^), 0.352 1/h (Kan^R^-Sm^R^), 0.279 1/h (*cymA*-*mtr*-Sm^R^) and 0.276 1/h (*cymA*-*mtr*-*fim*).

**

**

**Appendix S2: Biomass distribution and current density determined from BES cultivations of *E. coli* strain set**

Current density (J) determined at BES electrodes with four different *E. coli* strains from the data shown in Fig. 5 at 55 h past lactate addition. Biomass fractions in the BES were determined as cell dry weight suspended in solution (m_Diss. biom._) and as biomass adsorbed onto the electrode surface at the end of the 5-day BES experiment (m_Ads. biom._). The electrode-bound biomass was determined using an XS205 analytical scale (Mettler Toledo, USA) as weight loss of the dried carbon cloth resulting from overnight incubation in 1.0 M NaOH. The dry weight of suspended biomass was calculated from the OD_595_ using the experimentally determined conversion factor α = 0.25 ± 0.06 mg/mL. To estimate the number of electrode bound cells, the dried, electrode bound biomass was compared to the dry weight of *E. coli* cultures with a defined OD_595_. At the beginning of BES experiment, 16.2 mg cell dry weight was added to the BES. The shown data were determined from triplicate measurements.

|  | Kan^R^-Sm^R^ | *fim*-Kan^R^ | *cymA*-*mtr*-Sm^R^ | *cymA*-*mtr*-*fim* |
| --- | --- | --- | --- | --- |
| J [µA/cm^2^] | 1.07 ± 0.13 | 0.65 ± 0.14 | 4.01 ± 1.32 | 1.70 ± 0.16 |
| m_Diss. biom._ [mg] | 4.69 ± 0.85 | 4.22 ± 1.56 | 5.42 ± 1.43 | 6.46 ± 1.56 |
| m_Ads. biom._ [mg] | 3.69 ± 0.50 | 3.31 ± 0.21 | 7.97 ± 1.64 | 10.28 ± 1.66 |

**Appendix S3: Plasmids used in this study**

| **Plasmid name** | **Description** |
| --- | --- |
| pCR2.1-TOPO | Kan^R^ and Amp^R^. Supplied by Thermo Fisher Scientific (USA). |
| pCR2.1-TOPO+PCR4-c6 | Derivative of pCR2.1-TOPO containing *fimAICD*. |
| pCR2.1-TOPO+PCR5-c6 | Derivative of pCR2.1-TOPO containing *fimDFGH*. |
| pCDFDuet-1 | Sm^R^*.* Supplied by Merck KGaA (Germany). |
| pEC86 | Chl^R^. pACYC184 construct containing *E. coli* cytochrome *c* maturation genes *ccmA-H* downstream of *tet* promoter ([Arslan *et al.*, 1988](#_ENREF_1)). |
| pMil20 | Derivative of pCDFDuet-1 containing *fimAICDFGH* downstream of T7/*lac* promoter. Depicted in Supplement S2. |
| pSB1ET2 | Insert-free derivative of plasmid pET30a+ ([Jensen *et al.*, 2010](#_ENREF_3)). |
| pSH2 | Chl^R^. Derivative of pACYC184 containing a 11.2-kb chromosomal DNA fragment of the clinical *E. coli* isolate J96. Provided by Paul Orndorff (North Carolina State University, U.S.A) ([Orndorff and Falkow, 1984](#_ENREF_5)). |
| I5049 | Kan^R^. pET30a+-Construct containing *cymAmtrCAB* downstream of T7/*lac* promoter ([Jensen *et al.*, 2016](#_ENREF_4)). |

**Specific Bibliography (remaining references are included in main document)**

Arslan, E., Schulz, H., Zufferey, R., Künzler, P., and Thöny-Meyer, L. (1988) Overproduction of the *Bradyrhizobium japonicum* *c*-type cytochrome subunits of the *cbb3* oxidase in *Escherichia coli*. *Biochem Biophys Res Commun* **251**: 744-747.

| **Appendix S4: Map of vector pMil020**  The construct pMil020 is based on the expression vector pCDFDuet-1 and contains the structural *fim* gene cluster *fimAICDFGH*, a streptomycine resistance marker (*aadA*) under the control of IPTG-inducible promoter T7/*lac*, the CloDF13 origin of replication as well as a copy of gene expression regulator gene *lacI*. Genes are shaded in order to mark their expression mode, e.g., constitutively expressed genes are shown in black and genes under the control of the *lac* repressor are highlighted by grey coloring. The restriction sites flanking the ligated fragments are indicated.  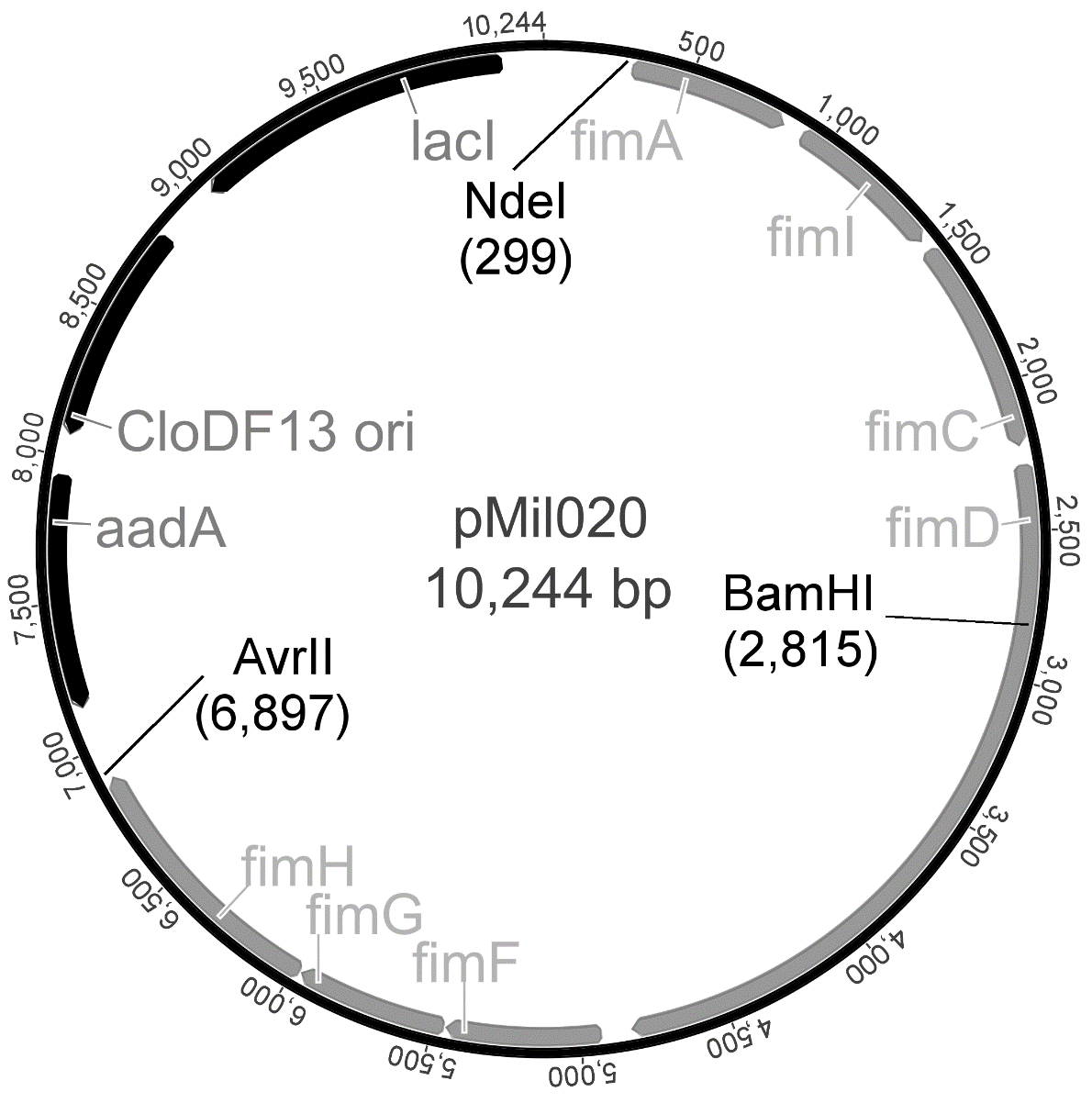  **Appendix S5: Primers used in this study**   \| **Primer name** \| **Nucleotide sequence (5' → 3')** \| \| --- \| --- \| \| AvrII_fimH_rv1 \| GAT CAC CCT AGG TTA TTG ATA AAC AAA AGT CAC GCC \| \| NdeI_fim_fw1 \| TTA GAG CAT ATG AAA ATT AAA ACT CTG GCA ATC G \| \| SH2_27_rv \| CAG CCT GGA ATT GCT CAT CG \| \| SH2_24_fw \| ATT GTT CCC TGC CTG ACA CG \| |
| --- | --- | --- | --- | --- | --- | --- | --- | --- | --- | --- |

**Appendix S6 Supplemental Material Information 1: Composition of M1 medium**

The M1 growth medium was prepared according to a protocol published by Bretschger *et al.* ([Bretschger *et al.*, 2007](#_ENREF_2)) by mixing 970 mL M1 base with 10 mL mineral supplement (MS), 10 mL vitamin supplement (VS) and 10 mL amino acid supplement (AAS). The M1 base contained 15.1 g PIPES (c_Final_ = 50 mM), 3.4 g NaOH, 1.5 g NH_4_Cl (c_Final_ = 28.04 mM), 0.1 g KCl (c_Final_ = 1.34 mM), 0.6 g NaH_2_PO_4_ · H_2_O (c_Final_ = 4.35 mM) and 2.0 g casamino acids dissolved in 950 mL ddH_2_0. The pH of the M1 base was adjusted to 7.4 and the solution was sterilized by autoclaving. The VS contained 2.0 mg biotin (c_VS_= 8.2 µM), 2.0 mg folic acid (c_VS_ = 4.5 µM), 10.0 mg pyridoxine-HCl (c_VS_= 48.6 µM), 5.0 mg thiamine-HCl · H_2_O (c_VS_ = 14.1 µM), 5.0 mg nicotinic acid (c_VS_= 40.6 µM), 5.0 mg d-(+)-pantothenic acid calcium salt (c_VS_= 21.0 µM), 0.1 mg cyanocobalamin (c_VS_= 74 nM), 5.0 mg *p*-aminobenzoic acid (c_VS_= 36.5 µM), 5.0 mg α-lipoic acid (c_VS_= 24.2 µM) dissolved in 1.0 L of ddH_2_O at a pH adjusted to 7.0. The AAS contained 2.0 g l-glutamic acid (c_AAS_= 14 µM), 2.0 g l-arginine (c_AAS_= 11 µM) and 2.0 g l-serine (c_AAS_= 15 µM) dissolved in 1.0 L of ddH_2_O at a pH adjusted to 7.0. The MS was prepared by first dissolving 1.5 g nitrilotriacetic acid (c_MS_ = 7.8 mM) in 800 mL ddH_2_O and the pH was adjusted to 8.0 with NaOH. Then, 3.0 g MgSO_4_ · 7 H_2_O (c_MS_ = 12.2 mM), 0.50 g MnSO_4_ · H_2_O (c_MS_ = 3.0 mM), 1.0 g NaCl (c_MS_ = 17.1 mM), 0.10 g FeSO_4_ · 7 H_2_O (c_MS_ = 0.36 mM), 0.10 g CaCl_2_ (c_MS_ = 0.68 mM), 0.10 g CoCl_2_ · 6 H_2_O (c_MS_ = 0.42 mM), 0.13 g ZnCl_2_ (c_MS_ = 0.95 mM), 10 mg CuSO_4_ · 5 H_2_O (c_MS_= 40 µM), 10 mg AlK(SO_4_)_2_ · 12 H_2_O (c_MS_ = 21 µM), 10 mg H_3_BO_3_ (c_MS_= 0.16 mM), 25 mg Na_2_MoO_4_ · 2 H_2_O (c_MS_ = 0.10 mM), 24 mg NiCl_2_ · 6 H_2_O (c_MS_= 0.10 mM) and 25 mg Na_2_WO_4_ · 2 H_2_O (c_MS_ = 76 µM) were added, the pH adjusted to 7.0 and H_2_O added to a final volume of 1.0 L. VS, AAS and MS were filter-sterilized, VS and AAS were stored at 4 ºC. VS was kept in the dark.

**Specific Bibliography**

Bretschger, O., Obraztsova, A., Sturm, C.A., Chang, I.S., Gorby, Y.A., Reed, S.B.*, et al.* (2007) Current production and metal oxide reduction by *Shewanella oneidensis* MR-1 wild type and mutants. *Appl Environ Microbiol* **73**: 7003-7012.
